# Parental origin of deletions and duplications – about the necessity to check for cryptic inversions

**DOI:** 10.1186/s13039-018-0369-1

**Published:** 2018-03-09

**Authors:** Thomas Liehr, Isolde Schreyer, Alma Kuechler, Emmanouil Manolakos, Sylke Singer, Andreas Dufke, Kathleen Wilhelm, Tereza Jančušková, Radek Čmejla, Moneeb A. K. Othman, Ahmed H. Al-Rikabi, Kristin Mrasek, Monika Ziegler, Stefanie Kankel, Katharina Kreskowski, Anja Weise

**Affiliations:** 1 Jena University Hospital, Institute of Human Genetics, Friedrich Schiller University, Postfach D-07740, Jena, Germany; 2 Center for Ambulant Medicine, Jena University Hospital, Jena, Germany; 3 Institut für Humangenetik, Universitätsklinikum Essen, Essen, Germany; 4 Access to Genome, ATG Labs, Athens, Greece; 5 Institut für Medizinische Genetik und angewandte Genomik, Tübingen, Germany; 6 Synlab czech s.r.o., synlab genetics s.r.o, Praha, Czech Republic

**Keywords:** Copy number variants (CNVs), Microdeletion/microduplication syndromes (MMSs), Three color fluorescence in situ hybridization (FISH), Inversion, Deletion, Duplication

## Abstract

**Background:**

Copy number variants (CNVs) are the genetic bases for microdeletion/ microduplication syndromes (MMSs). Couples with an affected child and desire to have further children are routinely tested for a potential parental origin of a specific CNV either by molecular karyotyping or by two color fluorescence in situ hybridization (FISH), yet. In the latter case a critical region probe (CRP) is combined with a control probe for identification of the chromosome in question. However, CNVs can arise also due to other reasons, like a recombination-event based on a submicroscopic, cryptic inversion in one of the parents.

**Results:**

Seventy-four patients with different MMSs and overall 81 CNVs were studied here by a novel three color FISH approach. The way how three locus-specific probes are selected (one is the CRP and two are flanking it in a distance of 5-10 Mb) enables to detect or exclude two possible parental conditions as origins of the CNV seen in the index: (i) direct parental origin of the CNV (deletion or duplication) or (ii) a parental cryptic inversion. Thus, for overall 51/81 CNVs (63%) a parental origin could be determined. 36/51 (70.5%) inherited the CNV directly from one of the parents, but 15/51 (29.5%) were due to an exclusively by three color FISH detectable parental inversion. A 2:1 ratio of maternal versus paternal inheritance was found. Also almost two times more male than female were among the index patients.

**Conclusion:**

The new, here suggested three color FISH approach is suited for more comprehensive parental studies of patients with MMS. The detection rate for parental origin was increased by 140% in this study. Still, for 30/81 cases (37%) no reason for the ‘de novo’ MMS in the affected index patient could be found by the here suggested FISH-probe set.

**Electronic supplementary material:**

The online version of this article (10.1186/s13039-018-0369-1) contains supplementary material, which is available to authorized users.

## Background

Copy number variants (CNVs) are a topic of highest interest in research and diagnostics [1]. Gain or loss of submicroscopic regions can either lead to clinical signs and symptoms [1, 2], suspected to be associated with diseases [1–3] or, according to present knowledge, just be variations without any significant meaning for the individual carrier [1, 4].

However, the way how CNVs change their size if they are passed from one generation to the next, i.e. how specific DNA-regions are lost or amplified within a CNV, is not completely understood, yet. Unequal crossing over events in low copy repeat regions [1, 5, 6], or complicated repair mechanisms after DNA-break and/or replication stress, like non allelic homologous recombination (NAHR), microhomology-mediated break-induced replication (MMBIR), or even chromothripsis are discussed [1, 6–8]. Another idea for CNV-formation related to MMBIR is based on the fact that regions involved in microdeletion/microduplication syndromes (MMSs) [2] can be flanked by repetitive elements being identical to each other, as first described by Jim Lupskis group for a ~ 1.4 Mb region in 17p12 including the *PMP22* gene [9]. Such a genetic environment may cause unequal crossing over during meiosis (or even mitosis [10, 11]), and thus deletion or duplication; i.e. a de novo CNV may arise in gametes or somatic cells. Recently, evidence was provided that at least in a subset of MMSs their corresponding critical regions (CRs) are flanked by DNA-stretches with sequence identity of 10-200 kb in size [12]. Besides, it has been shown that these CRs can be ‘inverted’ in one of the parents of a patient (e.g. [13, 14]), making a deletion or duplication more likely due to inversion loop formation [15]. Surprisingly, fathers or mothers of an MMS-patient may also be carrier of the identical deletion or duplication as the index patient, but without showing any, or only minimal symptoms of the corresponding syndrome [2]. This has been explained by the so-called two-hit model, suggesting a second large CNV only present in the patient and not in the parent [12, 16], or by a disease causing mutation in the not-deleted, corresponding relevant gene copy [17].

**Fig. 1:**
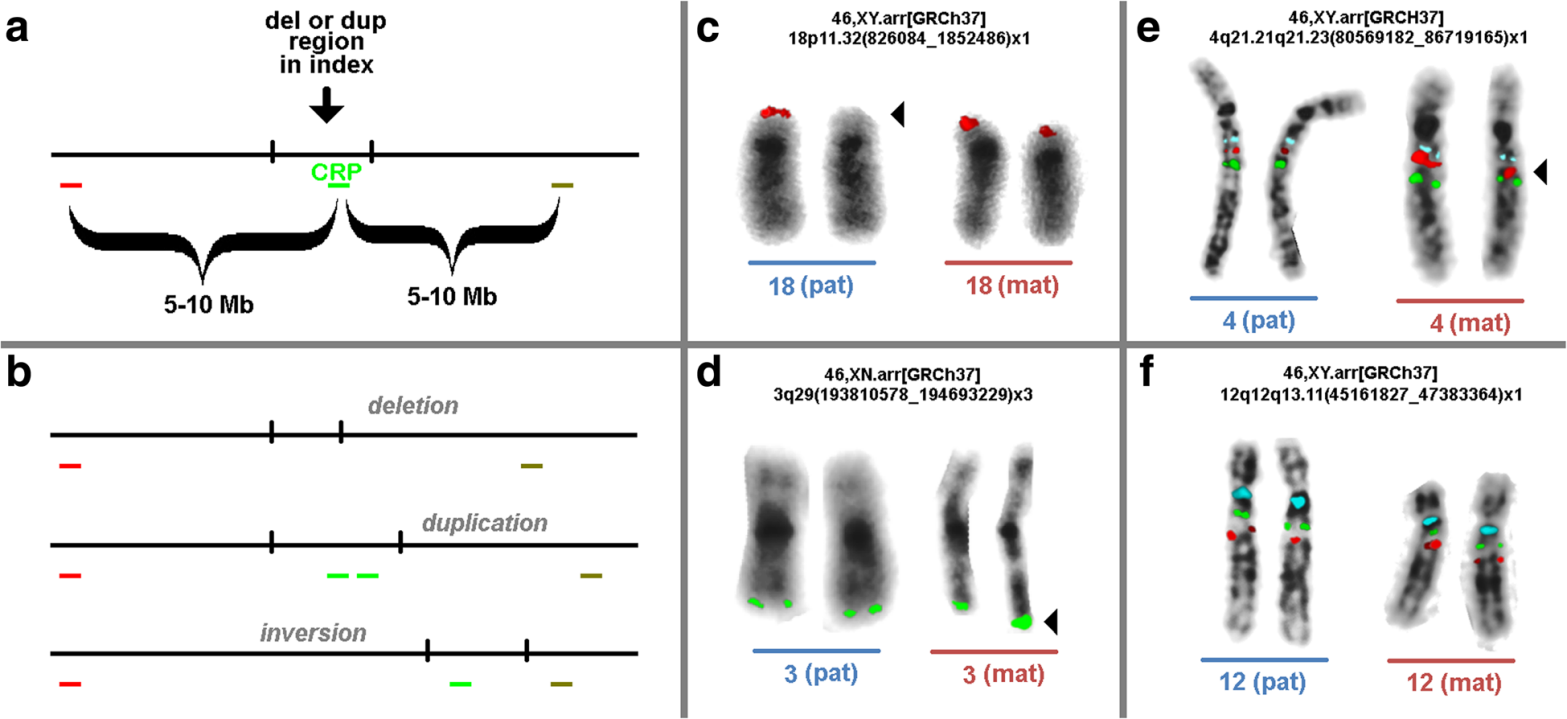
**a** Scheme of the probe design to detect deletions, duplications or inversions in the parents of an index patient with submicroscopic deletions or duplications. Abbreviations: CRP = critical region probe; Mb = megabasepair. **b** Schematic depiction of expected results. **c**-**f** Examples of parents of the index patients cases 18, 23, 32 and 54. The corresponding array-CGH results of the index patients are given in the figures; for the applied locus-specific probes see Additional file 1: Table S1. In Figs. **c** and **d** only the probe for the critical regions are depicted. Aberrant chromosomes are highlighted by arrowheads. **c** Case 18 had paternally derived deletion, as clearly visible. **d** Case 23 had a maternally derived duplication; the size of the duplication is too small to lead to two separated signals on the derivative chromosome 3, however, the signal-size and –intensity is clearly doubled compared to signal on the homologous. **e** A cryptic maternal inversion is depicted for case 32, as visible by the shift of the signal of the critical region probe. **f** No alterations could be detected in the parents of case 54; thus a ‘de novo’ formation of the copy number variant (CNV) in the index patient is suggested; as outlined in the text ‘de novo’ stands here for real de novo cases and such which may be based other (smaller) cryptic rearrangements not detectable by the here applied probe set

Here we analyzed parents of 74 index patients with different MMSs. A novel molecular cytogenetic approach was applied to detect or exclude two possible parental conditions as origins of the disease, as seen in the index: (i) direct parental origin of the CNV (deletion or duplication) or (ii) a parental cryptic inversion. Therefore we applied one locus-specific probe directly from the affected region (critical region probe = CRP) and flanked it with two probes, located between 5 and 10 Mb proximal and distal to the CRP. The analysis was done using a three color-fluorescence in situ hybridization (FISH) approach (Fig. 1). As 74 families and 81 CNVs were studied overall (Table 1 and Additional file 1: Table S1), a first approximation could be achieved on the frequencies of parental origin versus ‘de novo’ formation of an MMS. However, cases considered as formed ‘de novo’ still may be due to NAHRs or inversions, which are too small to be detected by the here suggested FISH-probe set, like listed [4] and discussed elsewhere [18, 19].

## Methods

### Samples

Parents of 74 index patients as summarized in Table 1 and Additional file 1: Table S1 were studied. All index patients suffered from developmental delay and or/dysmorphic features, and each of them had one up to two microdeletions and/or microduplications (Table 1 and Additional file 1: Table S1), as characterized by molecular karyotyping and/or standard GTG-banding elsewhere. Parents were clinically and cytogenetically normal. Chromosomes were prepared from cultured peripheral T-lymphocytes according to standard procedures [20]. Slides for metaphases FISH (see below) were produced following the air-drying protocol [21].

**Table 1:** Seventy-four families with affected children having deletion or duplication in one or two chromosomal regions included in this study are listed here. Overall 81 copy number variations (CNVs) distributed on all human chromosomes apart from #11 and Y-chromosome were studied. Cases with two CNVs were numbered as A and B, i.e. cases 11, 13, 14, 17, 19 and 46. For the index patient the chromosomal region affected, the mode of inheritance of the CNV (origin) and the gender are given here; for more details see Additional file 1: Table S1

Case number	Chromosomal region affected	Origin	Gender carrier	Case number	Chromosomal region affected	Origin	Gender carrier
1	2q21.1q21.3	del(mat/pat)cons.	n.a.	37	7p12.3p14.1	inv(mat)	m
2	12q15q21.2	del(mat)	f	38	4q13.3q22.1	inv(mat)	f
3	15q11.2	del(mat)	f	39	16p11.2	inv(mat)	f
4	6q14.3q15	del(mat)	f	40	17q21.31q21.31	inv(mat)mos	n.a.
5	6q22.33	del(mat)	m	41	2q23.1q23.2	inv(mat)mos	m
6	Xp22.33	del(mat)	m	42	7q32.3q33	inv(pat)	n.a.
7	12p12.3p12.3	del(mat)	m	43	7q31.32q32.2	inv(pat)	m
8a	4q13.2q21.21	del(mat)	m	44	6q21q22.31	inv(pat)	m
9	16p11.2	del(mat)	m	45	15q26.1q26.3	inv(pat)	m
10	2p16.3	del(mat)	f	46a	2p14	inv(pat)	m
11a	16p13.11	del(mat)	n.a.	47	15q13.2-q13.3	de novo	n.a.
11b	16p13.11	del(mat)	n.a.	48	8q24.3q24.3	de novo	n.a.
12	1q43q44	del(mat)	n.a.	49	19p13.2p13.3	de novo	m
13a	16p12.2	del(mat)	m	50	1p32.1p31.1	de novo	f
14a	14q12	del(mat)mos	n.a.	51	7q31.1q31.1	de novo	f
14b	15q11.2	del(mat)mos	n.a.	52	14q12	de novo	n.a.
15	7q11.23q21.11	del(pat)	f	53	16p11.2	de novo	n.a.
16	1q21.1	del(pat)	m	19b	15q11.2q13.1	de novo	f
17a	16p13.11	del(pat)	n.a.	54	12q12q13.11	de novo	m
17b	16p11.2	del(pat)	n.a.	55	13q22.2q31.1	de novo	f
18	18p11.32	del(pat)	m	56	10q22.3q23.2	de novo	n.a.
19a	2q13	del(pat)mos	f	57	7p15.3p15.2	de novo	m
20	7q22.1	del(pat)mos	m	58	6q13q15	de novo	n.a.
21	15q11.2	dup(mat)	n.a.	59	4q21.22q22.1	de novo	f
22	5q11.1q11.2	dup(mat)	n.a.	60	4q35.2	de novo	n.a.
8b	22q12.3q13.2	dup(mat)	m	61	3q26.3q27.3	de novo	n.a.
23	3q29	dup(mat)	n.a.	62	6q21q22.33	de novo	m
24	3q29	dup(mat)	n.a.	63	3p14.1p12.3	de novo	n.a.
25	19p13.3p13.3	dup(mat)	m	64	12q15q21.1	de novo	f
26	22q11.21	dup(mat)mos	m	65	16q24.1q24.2	de novo	n.a.
27	7q34q36.3	dup(mat)mos	n.a.	66	7p15.3	de novo	n.a.
28	4q13.1	dup(pat)	n.a.	67	9q22.31q22.33	de novo	n.a.
29	7q31.31q31.33	dup(pat)	f	68	16q24.1q24.3	de novo	n.a.
30	4q25	dup(pat)	n.a.	69	2q31.1	de novo	n.a.
31	13q12.13	dup(pat)	n.a.	70	21q22.12q22.2	de novo	n.a.
13b	8p23.1p22	dup(pat)	m	71	5q35.2q35.3	de novo	n.a.
32	4q21.21q21.23	inv(mat)	m	72	4q21.1q21.21	de novo	n.a.
33	20p12.3	inv(mat)	n.a.	73	10q11.22q11.23	de novo	n.a.
34	1p36.13p16.11	inv(mat)	n.a.	46b	2q31.2q31.3	de novo	m
35	17q12	inv(mat)	m	74	7q32.3q33	de novo	n.a.
36	17q21.31q21.31	inv(mat)	m				

*cons* consanguineous, *del* deletion, *dup* duplication, *inv.* inversion, *mat* maternal, *mos* mosaic, *pat* paternal

### Molecular cytogenetics

FISH was done according to standard procedures in a three color-FISH variant [22]. To straightforwardly identify the chromosomes of interest a corresponding whole chromosome painting or centromeric probe may be added in a fourth color. The principle scheme how the three color-FISH probe sets were constructed is shown in Fig. 1a. The expected results in case of deletion, duplication and inversion are depicted in Fig. 1b. Bacterial artificial chromosome (BAC) probes serving as CRP should not include regions of known CNVs, like e.g. used in studies detecting parental origin of chromosomes by FISH [23].

The probes as applied for each case are listed in Additional file 1: Table S1. Locus-specific BAC-probes were commercially available from BACPAC Resources Program (bacpac.chori.org) or available to the authors (via Synlab, Czech Republic). Probe labelling was done as previously reported [24]. Ten to Twenty metaphases were analyzed per case. Typical examples for FISH results are depicted in Fig. 1b-f.

## Results

**Fig. 2:**
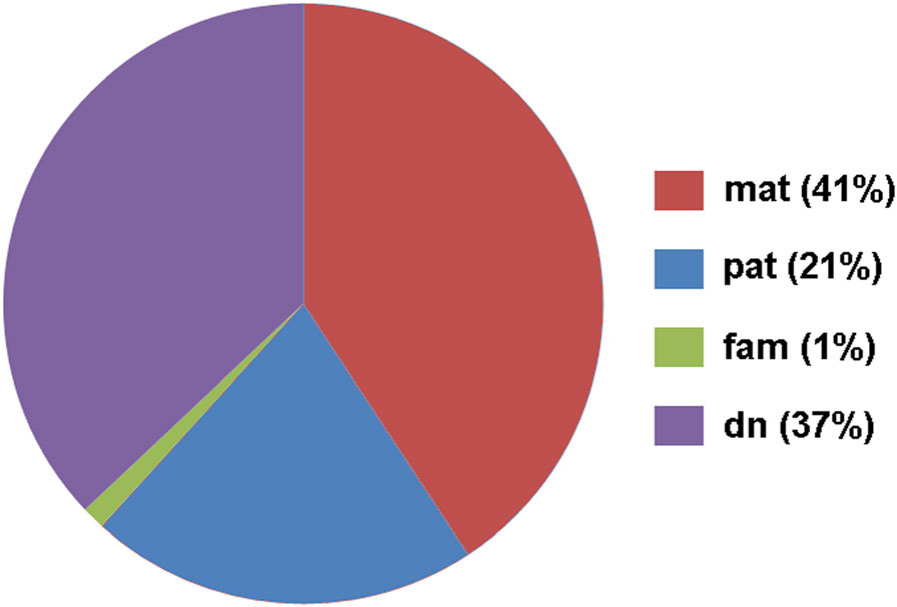
Parental origin of the 81 here studied copy number variants (CNVs). Abbreviations: dn = de novo; fam = familial; mat = maternal; pat = paternal

In this study the parental origin of 81 CNVs in 74 index patients was determined by a new three-color FISH approach (Fig. 1a and b). Therefore, one locus-specific probe mapped to the in the index patient affected region (critical region probe = CRP) was flanked by two probes, located between 5 and 10 Mb proximal and distal to the CRP. It could be convincingly shown that such kind of probe set enables simultaneous detection of deletions (Fig. 1c) or duplications (Fig. 1d), as well as cryptic, and in GTG-banding submicroscopic, parental inversions (Fig. 1e); the latter are visible as altered distances of the three probes to each other in the affected chromosome. Overall, the parental origin of potentially disease causing CNVs could be determined in 51 of 81 of the studied cases (63% - see Fig. 2). Also, there remained 37% of non-informative, here denominated as ‘de novo’ cases (Figs. 1f and 2).

**Fig. 3:**
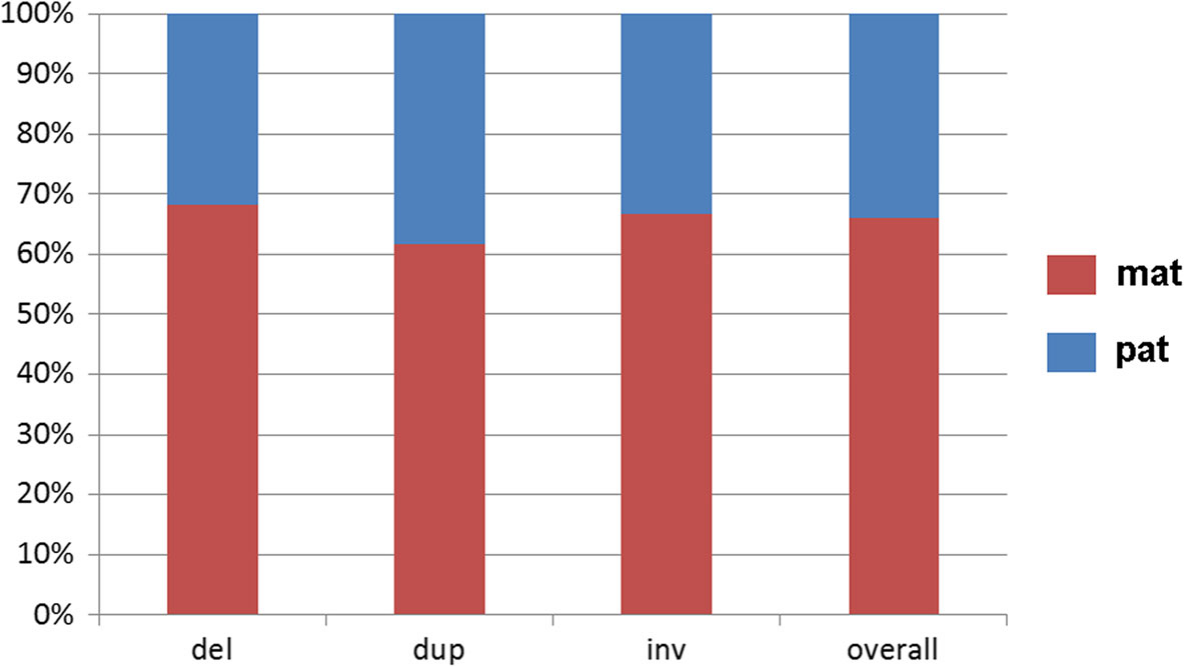
Parental derived CNVs of cases 2 to 46a were analyzed here for their maternal and paternal origin; 62 to 68% of the cases were maternally derived. Interestingly, in 8 cases mosaics of normal cells and cells with either maternal or paternal deletion, maternal duplication or maternal inversion were detected in the peripheral blood the tested parents (see Additional file 1: Table S1). Abbreviations: del = deletion; dup = duplication; inv. = inversion; mat = maternal; pat = paternal

Among the parentally derived cases there was one (case 1) with partial nullisomy 2q21.1 to 2q21.3 in the index patient. This was most likely due to consanguinity of the parents and heterozygosity of both for this CNV. The remainder 50 CNVs could be clearly attributed to be of maternal (33/50 cases = 66%) or paternal inheritance (17/50 cases = 34% - see Fig. 2). The obtained data is broken down more specifically in Fig. 3 for parental deletions, duplications or inversions. As shown in Table 1, four cases with deletion, and two cases each with duplication and inversion were mosaic in the transmitting parent.

**Fig. 4:**
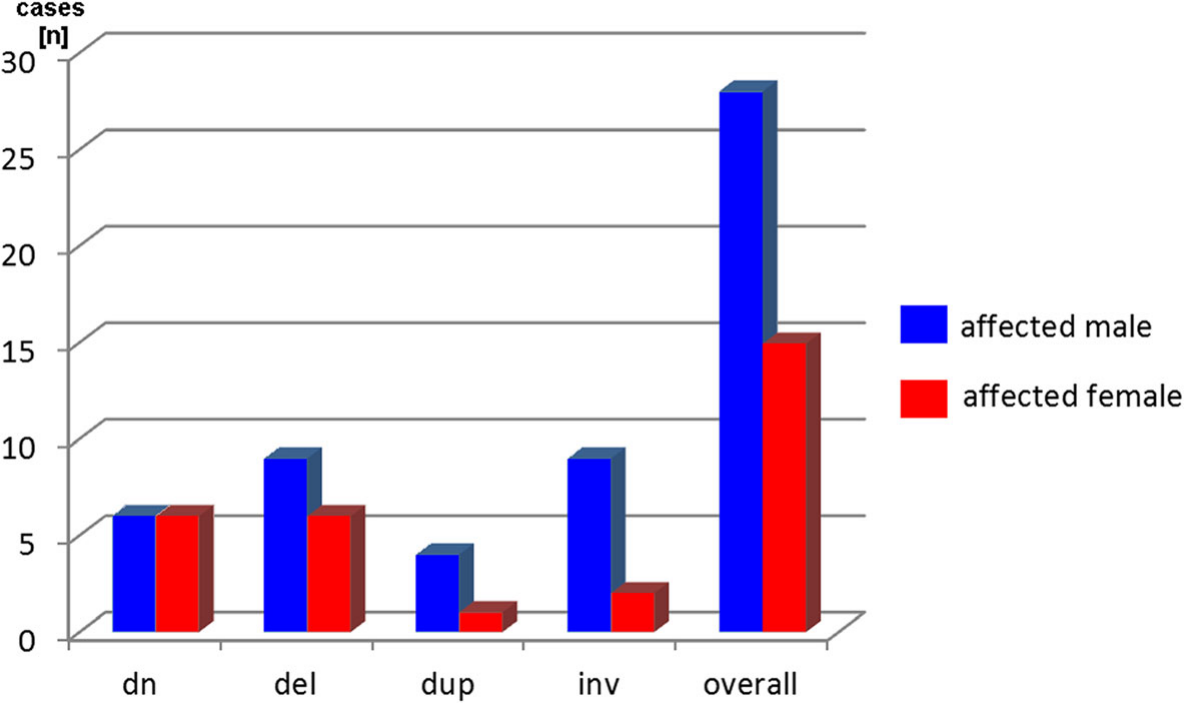
In 43 cases the gender of the affected index patient was available. Interestingly, overall more male than female were affected, if the CNV was inherited from one of the parents. Abbreviations: dn = de novo; del = deletion; dup = duplication; inv. = inversion

The gender of the index patient was only available in 32 of the 74 cases. Thus, it could be evaluated for 43 CNVs studied here. Interestingly, overall the male to female ratio of the index was 28 to 15; considering only ‘de novo’ cases the ratio was 1:1, and in inherited cases the ratio was 1.8 to 1 (Fig. 4).

**Fig. 5:**
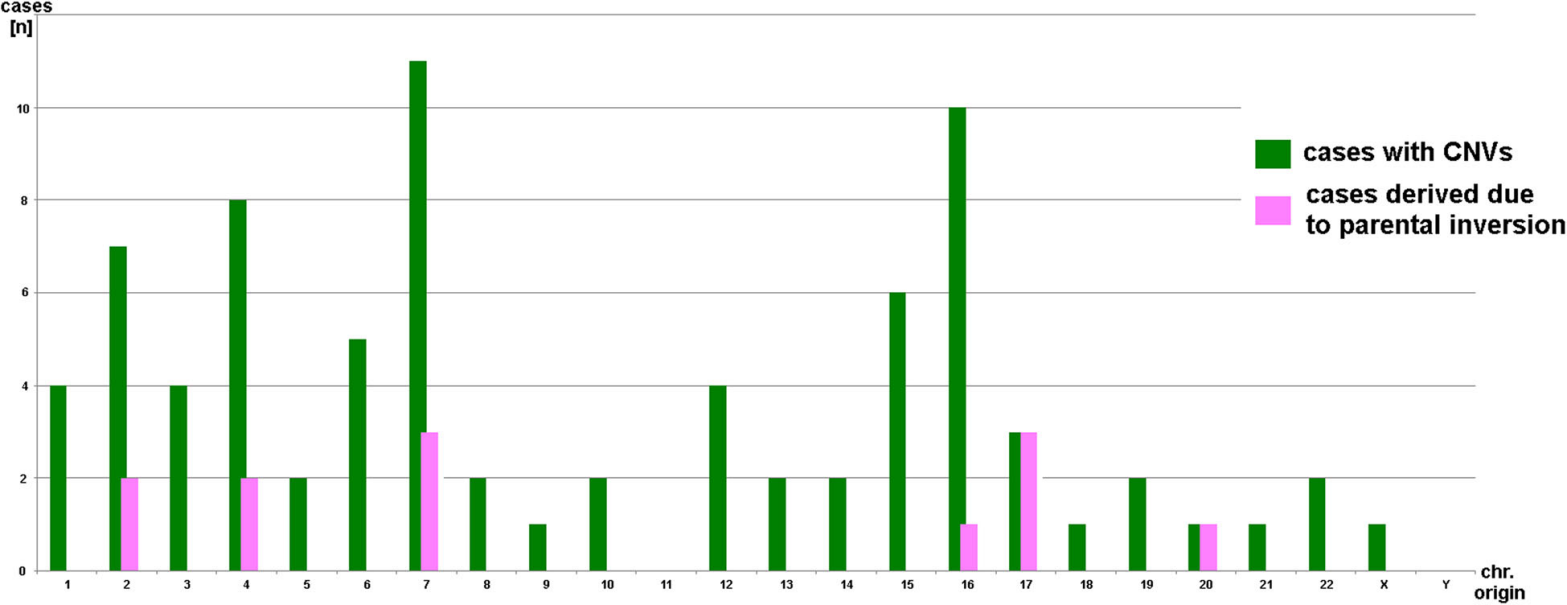
The 81 here studied copy number variants (CNVs) sotted according to their chromosomal origin; cases which are due to a parental submicroscopic inversion are highlighted in pink. Abbreviations: CNVs = copy number variants

An analysis for the chromosomal origin of the CNVs (Fig. 5) revealed that they derived from practically all chromosomes. 15/81 CNVs were due to parental inversions (18.5%) and those were detected on 6/24 chromosomes (25%). However, 3/11 CNVs studied for chromosome 7, and 3/3 CNVs studied for chromosome 17 were due to parental inversions. Finally, CNVs resulting from detectable parental inversions were between 545,601 bp (case 39) and 17,223,229 bp (case 38) in size. As the corresponding inversions may provide just only one of their breakpoints to the resulting CNV, the underlying inversions may have been larger or smaller.

## Discussion

CNVs may arise by different mechanisms as outlined above [1]. To provide information for affected families about the repetition risk in a following pregnancy, yet the only routinely offered studies for a possible parental origin of a CNV are either molecular karyotyping [25] or two-color-FISH using one CRP and one control probe; the latter is applied to identify the chromosome of interest [10]. Thus, yet only parentally derived deletions or duplications may be detected, but no structural changes. Even though single studies showed that it is necessary in MMS like Angelman/Prader-Willi [13] Williams-Beuren [14] and Sprintzen velocardiofacial syndrome [26] also to check for potentially, in the offspring disease causing inversions, no systematic studies in other MMSs were undertaken, yet. This gap was closed by the present study using a simple three-color-FISH probe set, as suggested here in Fig. 1a, which may be applied in each individual MMS case, as long as a CRP and flanking probes are available.

Thus, the detection rate of a proven parental origin for an MMS can be drastically enhanced. Traditionally, only using FISH with a CRP and a control probe or molecular karyotyping, parental origin of the deletion or duplication would have been solved in the present cohort for only 36/81 CNVs (44%). With the new, here presented approach 15 additional CNVs could be attributed to a parental origin (i.e. an inversion) raising the detection rate up to 63%. The remainder 30 cases, denominated here as ‘de novo’, could not be resolved with this probe set. They still may be due to NAHR, i.e. processes during gametogenesis like observed in Charcot-Marie-Tooth disease type 1A (CMT1A) [9, 10], or also be based on smaller inversions not detectable by this kind of metaphase-FISH directed probe set, but rather by interphase-FISH [14]. Interestingly, for all three groups (deletion, duplication and inversion) examples were found, which were present only in mosaic state in the transmitting parent. This suggests most likely a postzygotic origin of the rearrangement in the parent; still, also reversion could be considered. Cases like that are already reported e.g. for CMT1A and have been shown to go together with a less severe symptomatic [10], which goes together well with the fact that all parents in this study were considered as asymptomatic. Furthermore, mosaic conditions are known to be variable in different tissues of the body [27]; thus, for all parental tests presently available it has always to be considered that gonadal mosaicism can never be excluded [28, 29].

The observed partial nullisomy 2q21.1 to 2q21.3 in the index patient of case 1, which is most likely due to consanguinity and partial monosomy in both of the parents, also reminds of the possibility that in rare cases a uniparental disomy (UPD) may lead to an MMS with nullisomy or partial tetrasomy [30, 31]. For case 1 a UPD(2) was not excluded, as a biparental origin is here much more likely.

Interestingly, the parental origin of the CNVs showed the same 2:1 (maternal: paternal) ratio (Fig. 3) as e.g. known for the inheritance of small supernumerary marker chromosomes [32]. This normally is explained by higher tolerance of the female gamete producing system towards mistakes in the oocytes, than the testes have towards genetically defective sperm [32–34]. However, the gender ratio of the offspring of cases with parental origin of the CNV of 1:1.8 (female: male) (Fig. 4) is not as easy to explain. Besides a possible bias due to small numbers, it may reflect the fact that CNVs in female are better compensated than in male. Such effects are e.g. observed for patients with ‘large CNVs’, like in trisomy 13 or 18 [35]. Lion hypotheses and the female mosaic condition of the X-chromosome-inactivation of paternal and maternal copy are discussed to be involved here [36].

Finally, it is known that CNVs seem to be present more or less equally distributed over all human chromosomes [4]; for sure, when looking closer there are hot spots for them, as also reflected by different recombination rates of DNA-stretches with and without CNVs [15]. The chromosomal origin of the CNVs in this study provides no surprise here (Fig. 5). If the relative high rates of CNVs due to inversions in chromosomes 7 and 17 are meaningful or not, has to be ruled out by future studies. Interestingly for chromosome 17 an inversion polymorphism was reported recently [37].

## Conclusions

Overall, the here suggested new three-color-FISH approach is straightforward and can be universally applied for more comprehensive parental studies of patients with MMS. The detection rate can be increased by 140% as now also inversions and not only parental deletions and duplications can be detected, which might provide implications for genetic counselling, risk calculation for close relatives, as well as an option for prenatal testing. Still the here applied BAC-probesets are only suited for metaphase-FISH; for interphase-FISH distances of 2-5 Mb between CRP and flanking probes would be indicated [14], and BAC-probes not leading to background and cross-hybridization problems. Nonetheless, the here presented FISH probe set would also be able to detect the elsewhere reported but rare parental insertional balanced translocations as reason of CNV in the offspring [38].

## Additional file

Additional file 1: **Table S1.** Besides details listed in Table 1, karyotype of index patient after GTG-banding and array-CGH, and the locus-specific probes used for the molecular cytogenetic study of the parents of the index patient are provided. No detailed clinical data is given, as this is not of interest for this study; all patients were studied due to developmental delay and or/dysmorphic features. (XLSX 19 kb)

## Abbreviations

BAC: Bacterial artificial chromosome
CMT1A: Charcot-Marie-Tooth disease type 1A
CNVs: Copy number variants
CR: Critical region
CRP: Critical region probe
FISH: Fluorescence in situ hybridization
GTG: G-bands by trypsin using Giemsa
MMBIR: Microhomology-mediated break-induced replication
MMSs: Microdeletion/microduplication syndromes
NAHR: Nonallelic homologous recombination
UPD: Uniparental disomy
